# Genomic insights into Brevibacterium sediminis strain IMA_C3 isolated from an integrated mangrove aquaculture pond

**DOI:** 10.1099/acmi.0.000996.v4

**Published:** 2026-02-13

**Authors:** Yash, Anwesha Ghosh, Ajanta Dey, Milon Sinha, Nimai Bera, Sabyasachi Chakraborty, Punyasloke Bhadury

**Affiliations:** 1 Integrative Taxonomy and Microbial Ecology Research Group, Department of Biological Sciences, Indian Institute of Science Education and Research Kolkata, Mohanpur-741246, Nadia, WB, India; 2 Centre for Climate and Environmental Studies, Indian Institute of Science Education and Research Kolkata, Mohanpur-741246, Nadia, WB, India; 3 Nature Environment and Wildlife Society, 10, Chowringhee Terrace, Kolkata-700020, WB, India

**Keywords:** *Brevibacterium*, mangrove litter, nitrogen, secondary metabolites

## Abstract

*Brevibacterium sediminis* strain IMA_C3, a Gram-positive bacterium, was isolated from an integrated mangrove aquaculture pond near the Sundarbans mangrove. The bacterium was isolated from mangrove leaf litter and grown on Luria-Bertani medium at a salinity of 20. Phylogenetic analysis based on 16S rRNA sequencing showed a 99.67% identity with *Brevibacterium linens* AE038-8 from the International Nucleotide Sequence Database Collaboration DNA databases (GenBank/DDBJ/ENA). Whole-genome sequencing was carried out using long-read sequencing on the Oxford Nanopore MinION platform, with genome annotation performed against the NCBI Reference Sequence Database and The Genome Taxonomy Database databases. The genome is ~4.1 Mb in size, with a G+C content of 64.59 mol%. Functional analysis of the genome revealed genes related to complex carbon utilization, nitrogen and phosphate metabolism and metal transport. Additionally, the genome encodes secondary metabolites, including ε-poly-l-lysine, ectoine, terpene and phenazine, which could have potential applications in controlling viral infections in indigenous shrimp populations within integrated mangrove aquaculture systems.

## Data Summary

The whole-genome sequence data are available in National Center for Biotechnology Information (NCBI) (BioProject accession number PRJNA1176022 and BioSample accession number SAMN44378832).

## Introduction

*Brevibacterium* is a Gram-positive bacterial genus and is the sole known representative of the family *Brevibacteriaceae* [1,2]. Members of this genus exhibit a wide range of morphological, physiological and biochemical traits [3]. *Brevibacterium* species are ubiquitous in nature and have been isolated from diverse environmental matrices, including soil (such as salt lakes and marine sediments), decomposed litter and various fermented foods such as dairy products. These bacteria are slender, rod-shaped, non-acid-fast organisms that undergo a characteristic rod-to-coccus morphological cycle (Irlinger *et al.* 2017) [4]. Species within this genus are known to produce enzymes such as proteinases and catalase, which often exhibit halotolerant properties. Notably, *Brevibacterium* can convert l-methionine into methanethiol (CH_₃_SH), a volatile organosulphur compound with significant industrial and ecological relevance [4]. These bacteria are mesophilic and taxonomically placed within the phylum *Actinomycetota* [2,5].

Mangrove litter dynamics play a crucial role in nutrient cycling, particularly in regulating major nutrient pools such as carbon, nitrogen and phosphate, as evident in estuarine coastal ecosystems, integrated mangrove aquaculture (IMA) and sustainable mangrove aquaculture fisheries [6,9]. Litter decomposition is primarily driven by both physical and biological agents [10]. Microbial communities, particularly bacteria, are vital for breaking down complex organic matter and facilitating nutrient turnover [4,10]. Therefore, the isolation and characterization of these bacteria are essential for understanding their ecological functions and for leveraging their potential in nutrient cycling within mangrove-associated ecosystems.

Among bacterial groups, *Brevibacterium* species are known for their role in balancing nutrient stoichiometry, particularly between nitrogen and phosphate, as well as for participating in metal metabolism within ecosystems [4,11, 12]. This group of micro-organisms has also shown significant potential in aquaculture, particularly in controlling the spread of white spot syndrome virus (WSSV) in indigenous tiger shrimp (*Penaeus monodon*) through the biosynthesis of silver (Ag) and gold (Au) nanoparticles [2,13]. Given their diverse applications and functional versatility, *Brevibacterium* spp. present promising opportunities for further isolation and investigation to enhance bioresource growth and productivity in aquaculture.

## Methods

### Sampling and isolation

Surface water samples and floating leaf litter of *Avicennia officinalis* L. were collected from an IMA pond located in Chaital, Minakha Block, North 24 Parganas, near the Sundarbans, India (C3; 22° 29ʹ 59.8ʺN, 88° 47ʹ56.2ʺE). The collected leaf litter was immediately transferred into a sterile 50 ml centrifuge tube (Tarsons, India) using a sterile tweezer. The sample was transported immediately under temperature-controlled conditions to the lab. Upon arrival, the surface of the leaf was washed with freshly prepared PBS at pH 7.4, and the washed extract was used as the inoculum. A volume of ~20 µl of the extract was spread onto Luria-Bertani agar plates prepared with a salinity of 20 to mimic environmental conditions of the collection site and subsequently incubated overnight at 37 °C. Visible colonies were isolated and re-streaked to obtain pure cultures. These pure colonies were examined under a bright-field microscope at 100× magnification (BX53, Olympus, Japan) following Gram staining to determine cell morphology and Gram reaction. Gram staining revealed only one single pure Gram-positive colony that was aseptically transferred into a liquid growth medium with the same nutrient and salinity conditions. All culture work was performed in parallel with a negative control. This isolate has been designated as IMA_C3.

### Genomic DNA extraction

Genomic DNA (gDNA) was extracted from an overnight-grown culture using a modified phenol–chloroform protocol based on established methods [14,15]. Briefly, cells were pelleted by centrifugation at 6,000 r.p.m. for 5 min. The resulting pellet was resuspended in lysis buffer containing 50 mM Tris(hydroxymethyl)aminomethane Hydrochloride (Tris-HCl), 20 mM EDTA, 400 mM Sodium Chloride (NaCl), 750 mM sucrose (each 100 µl) and 10 µl of 10% SDS (Merck, India). The mixture was incubated in a water bath at 50 °C for 1 h to allow cell lysis.

Subsequently, 5 µl of Proteinase K (10 mg ml^−1^; HiMedia, India) was added, followed by incubation at 55 °C for 2 h. Then, 10 µl of lysozyme (10 mg ml^−1^; Merck, India) was added and incubated at 37 °C for another 2 h. DNA was extracted by adding an equal ratio (1:1) of phenol and chloroform (Merck, India), gently mixing for 5 min, and allowing the mixture to stand for 15 min, followed by centrifugation at 16,000 relative centrifugal force (r.c.f.) for 12 min, and the aqueous phase containing the gDNA was carefully collected.

The gDNA precipitation was carried out by adding 50 µl of 3 M sodium acetate (Merck, India) and 750 µl of absolute ethanol (Merck, Germany), followed by incubation at 20 °C for 12 h. The precipitated gDNA was recovered by centrifugation at 16,000 r.c.f. for 12 min and resuspended in 20 µl of 10 mM Tris-HCl (pH, 8.0).

The quality of the extracted gDNA was evaluated by electrophoresis on a 1% agarose gel stained with a fluorescent dye (ethidium bromide-EtBr; Merck, India), and the concentration (yield) was measured using a NanoDrop 2000c spectrophotometer (Thermo Fisher Scientific, USA).

### Genotypic identification

The isolate was identified using eubacterial primers Fc27 and Rc1492 [16] to amplify 16S rRNA, followed by Sanger sequencing. Raw chromatograms were manually inspected and curated using BioEdit (v7.7.1.0). Sequence identification was performed using the BLASTn algorithm against the GenBank/DDBJ/ENA databases. To determine the closest taxonomic affiliation, 16S rRNA gene sequences of related taxa were retrieved from the same databases and aligned using clustal-W [17]. The best-fit nucleotide substitution model for maximum-likelihood (ML) phylogenetic analysis was determined using jModelTest (v2.1.6) [18]. An ML phylogenetic tree was constructed in mega11 with 1,000 bootstrap replicates under the Tamura–Nei model [19].

Whole-genome sequencing was conducted using the Ligation Sequencing Kit (SQK-LSK109; Oxford Nanopore Technologies, United Kingdom), and sequencing was carried out on the MinION platform using long-read sequencing chemistry following the manufacturers’ protocol. Raw reads were quality filtered (Q>8) using Filtlong (v0.2.1), and adapters were removed using Porechop (v0.2.0), read length below 1,000. Filtered long reads were used for *de novo* genome assembly with Flye (v2.9.3) [20] in nano-raw mode with a single polishing iteration. Assembly quality was evaluated using QUAST (v5.2.0) [21], and genome completeness was assessed using CheckM [22]. The draft genome was annotated using Prokka (v1.14.6) [23], with the following parameters: minimum contig size 200 bp and an e-value cut-off 0.000001, and a circular genome map was generated and visualized using Proksee (https://proksee.ca/) [24].

### Genome data processing

Whole-genome sequence-based phylogenetic analysis was conducted using the Type (Strain) Genome Server (TYGS) (https://tygs.dsmz.de) [25]. Genome distances were estimated using the Genome BLAST Distance Phylogeny (GBDP) method, and a balanced minimum-evolution tree with branch support was constructed using FastME (v2.1.6.1), incorporating subtree pruning and regrafting post-processing [26]. Taxonomic classification, essential gene identification and average amino acid identity calculations were performed using the Microbial Genomes Atlas (MiGA) platform (http://microbial-genomes.org/) . Digital DNA–DNA hybridization (dDDH) values were obtained using the Genome-to-Genome Distance Calculator (GGDC v3.0) hosted on the DSMZ server [27]. Average nucleotide identity (ANI) was calculated using both Orthologous Average Nucleotide Identity [28] and the EzBioCloud platform (https://www.ezbiocloud.net/tools/ani) [29].

Biosynthetic gene clusters (BGCs) encoding natural products, also referred to as secondary metabolites, were identified using antiSMASH [30]. Antimicrobial resistance genes were identified and annotated using the Resistance Gene Identifier (RGI) module of the Comprehensive Antibiotic Resistance Database (CARD) (https://card.mcmaster.ca) [31]. Metal and metalloid resistance genes were predicted using the deepMRG tool [32].

The potential for complex carbon source utilization from mangrove litter, along with associated metabolic pathways, was assessed using the GhostKOALA–KEGG Mapper and PLaBAse [33,35]. Carbohydrate-active enzymes were predicted using the dbCAN3 server [36]. Additionally, BAGEL4 was employed to detect ribosomally synthesized and post-translationally modified peptides and bacteriocins in the draft genome [37]. The presence of Clustered regularly interspaced short palindromic repeats (CRISPR)-Cas systems was determined using the CRISPR-Cas Finder tool [38].

## Results and discussion

### Genome characteristics

**Fig. 1.**
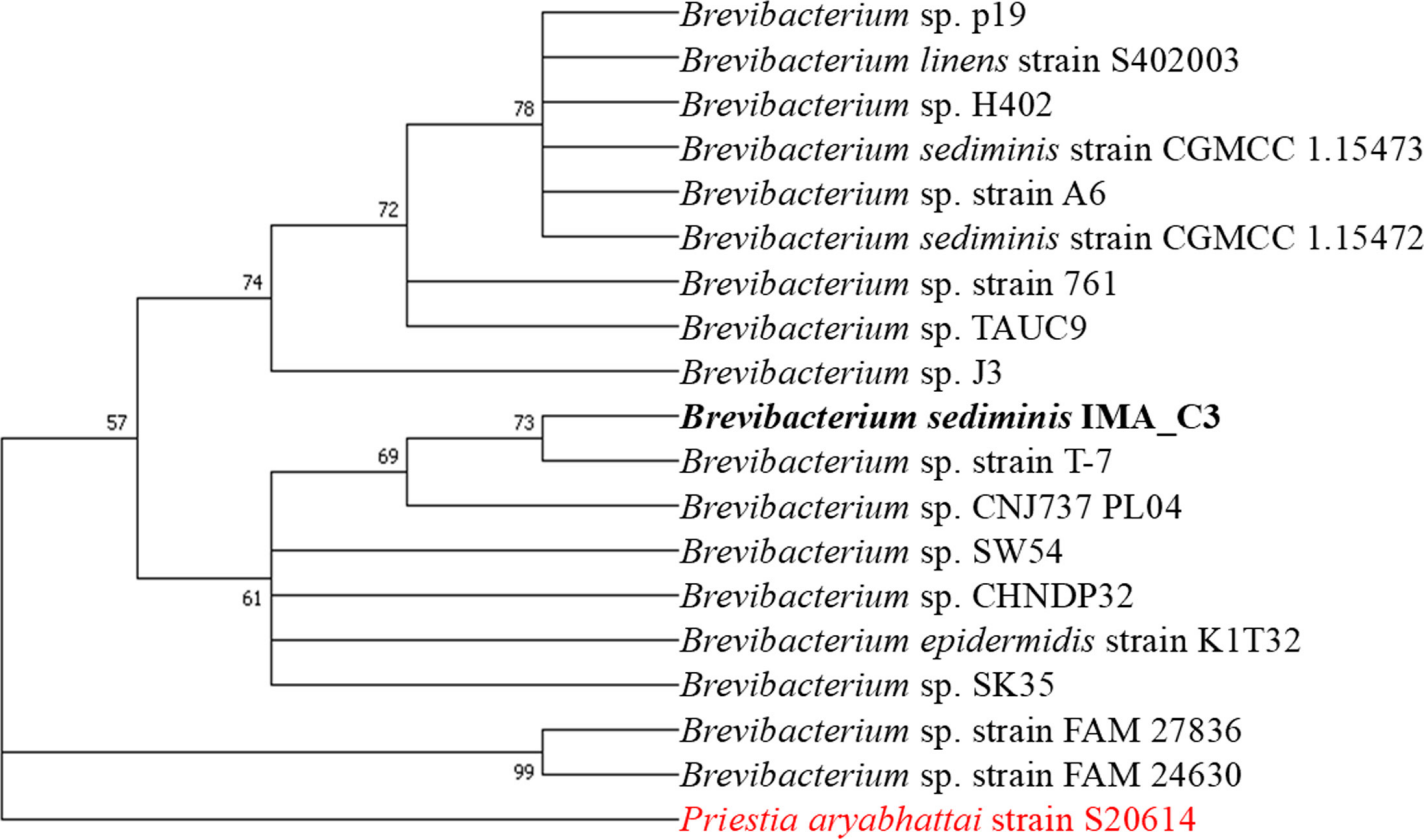
Phylogenetic analysis based on 16S rRNA sequence from ML-based phylogenetic tree with Tamura–Nei model (1,000 bootstrap replicates; bold font marks studied strain, and red font indicates out-group).

Gram staining revealed rod-shaped cells with an average size ranging from 0.55 to 0.71 µm in diameter and 2.42 to 3.15 µm in length. Based on 16S rRNA sequencing, the isolate was identified as a member belonging to the genus *Brevibacterium*; showing high sequence similarity with *Brevibacterium linens* S402003 (99.14%), *Brevibacterium sediminis* CGMCC (99.26%), *Brevibacterium oceani* INA5 (98.90%) and *Brevibacterium iodinum* BUS 3 (98.80%) being the closest relatives (Fig. 1).

**Fig. 2.**
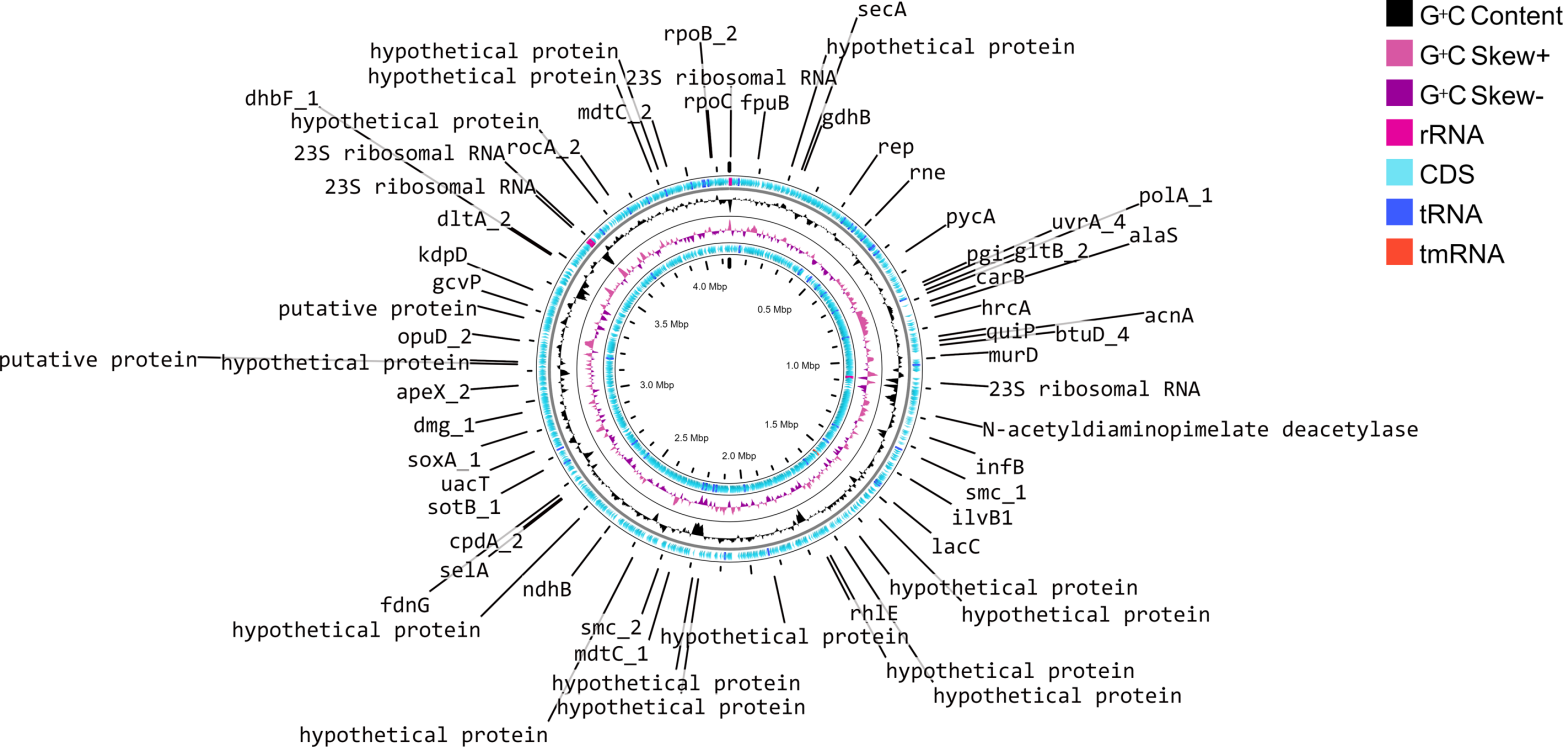
Genome map showing a circular genome, identified genes, G+C content and G+C skew (+/−) of *B. sediminis* strain IMA_C3.

**Fig. 3.**
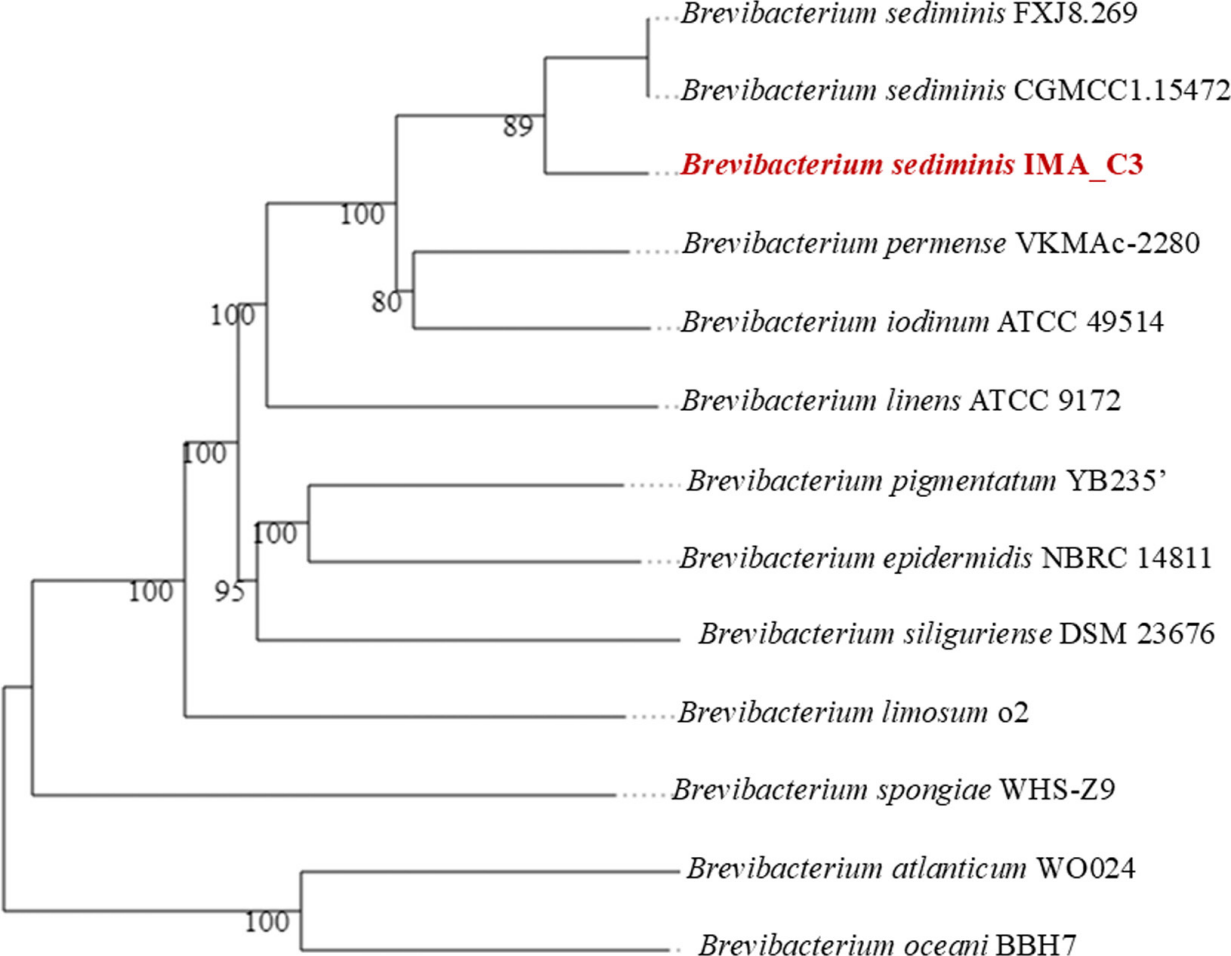
Phylogram showing phylogenetic relation of the isolate *B. sediminis* strain IMA_C3 with other known *Brevibacterium* sp. The red bold indicates the studied isolate.

The draft genome of the isolated strain meets the criteria for a high-quality genome, with a completeness score of 94.6% [39]. The genome was assembled into a single contig measuring ~4.1 Mb (4,141,684 bp), with a G+C content of 64.59 mol%. Genome annotation revealed the presence of 12 ribosomal RNA, 54 transfer RNA and 4,250 coding sequences (Fig. 2), and a circular genome map of *B. sediminis* strain IMA_C3 in comparison with the closest strains of the genus *Brevibacterium* (Fig. S1, available in the online Supplementary Material). Detailed genome assembly statistics, including G+C content percentage and the Table 1ne isolate within the *Brevibacterium* genus, based on pairwise comparisons using FastME 2.1.6.1 (Fig. 3). Calculation of dDDH and ANI is crucial for genome-wide comparisons of closely related taxa, as they rely on shared orthologous gene clusters to assess genomic relatedness. digital DNA-DNA hybridization (dDDH) and Average nucleotide identity based on BLASTn (ANIb) values indicate that this isolated bacterium belongs to the species *B. sediminis* strain IMA_C3 (Table 2).

**Table 1.** Draft genome assembly statistics of *B. sediminis* strain IMA_C3

Genomic feature	No. or length (bp)
G+C content (mol%)	64.59
No. of all contigs	1
No. of large contigs (>1,000 bp)	1
Contig N50	4,141,684
Contig N90	4,141,684
L50	1
L90	1
Total length	4,141,684

**Table 2.** Comparison of *B. sediminis* strain IMA_C3 with closely related species

Species	ANIb (%)	Distance	DNA–DNA hybridization (%)	G+C (mol%) difference
*B. oceani*	83.4	0.1599	27	0.74
*B. sediminis*	97.12	0.0232	80.2	0.07
*B. linens*	89.65	0.0976	40.6	0.19
*Brevibacterium pigmentatum*	90.17	0.096	41	0.06
*Brevibacterium epiderdimis*	89.47	0.1024	39.1	0.24
*Brevibacterium permenese*	93.36	0.0611	54.9	0.06
*Brevibacterium idionum*	92.77	0.0656	52.7	0.2

The genome was further studied to identify its genetic functions using genome annotation tools. The genome annotation of *B. sediminis* strain IMA_C3 showed the presence of functional genes potentially involved in nitrogen metabolism (e.g. *Ncds*, *Npd*, *GlnA*, *nitralase*, *GLUL*, *GudB*), phosphate transport (*PhoU*) and genes that could confer resistance to metals and metalloids, including *copA*, *copB*, *copC* and *copD* (copper resistance); *corA* (cobalt and magnesium); *czcR* (cobalt, zinc and cadmium) and *chrA* (chromium). The annotated genes are tabulated in Table S1. The genome also harbours BGCs that could encode ε-poly-l-lysine (100% similarity), ectoine (75%), terpene (57%) and phenazine (21%). The presence of the ε-poly-l-lysine BGC suggests potential for biocontrol applications, including the suppression of WSSV in shrimp, although this requires further validation.

Metabolic pathway analysis using Kyoto Encyclopedia of Genes and Genomes (KEGG) annotations revealed that 27.7% of pathways are associated with carbohydrate metabolism, while 6.01% relate to environmental information processing. The genome encodes β-glucosidase (EC:3.2.1.21), an enzyme involved in the breakdown of plant-derived polyphenols. Additionally, genes such as *galC*, encoding galactosylceramidase, are predicted to facilitate the depolymerization of complex carbon sources, including cellobiose, mannose and maltose from mangrove litter (Table S1). This breakdown transforms hydrolysable tannins into gallotannins, yielding monosaccharides that are accessible to organisms within the ecosystem.

The resistome of *B. sediminis* strain IMA_C3 includes genes such as *vanW* (28.57%), *sul4* (48.57%), *golS* (33.9%), *tetA* (30.77%), *nalD* (43.28%), *adeN* (47.17%) and *evgA* (26.94%), as predicted by the RGI database based on loose hits. These genes are associated with antibiotic efflux mechanisms conferring resistance to sulphonamides, fluoroquinolones, macrolides, tetracyclines and cephalosporins. Resistance may also arise from target-site modifications. Moreover, the genome contains genes linked to the degradation of polycyclic aromatic hydrocarbons, such as fluoranthene, naphthalene, xylene, acenaphthene, toluene and anthracene.

A CRISPR array was detected on contig 1 of the genome (4,141,684 bp), consisting of one spacer and a direct-repeat length of 36. The genome also includes genes associated with the production of linocin, a bacteriocin previously reported in *B. linens* [40].

Overall, this isolated bacterial strain encompasses a suite of functional traits indicative of multifaceted ecological significance and potential utility in applied environmental systems. Genomic analysis reveals the presence of a diverse array of genes associated with nutrient metabolism, particularly those involved in nitrogen and carbon cycling, as well as amino acid biosynthesis. These genetic capabilities suggest that the bacterium may contribute to nutrient eco-stoichiometry in aquatic ecosystems, a critical factor in maintaining water quality and ecological equilibrium in aquaculture environments.

Furthermore, the ability of the strain to biosynthesize essential amino acids position itself as a potential bio-augmentative agent capable of promoting the health and growth of cultured bioresources in coastal aquaculture ponds. These traits may reduce the dependency on chemical inputs such as antibiotics and enhance the sustainability of aquaculture practices. Nonetheless, while the *in vitro* functional potential is promising, validation through *in situ* or mesocosm-based studies is imperative to confirm its efficacy under field conditions. Future research should aim to characterize its interactions with native microbial communities, nutrient flux dynamics and its impact on host species health as well as productivity in integrated mangrove aquaculture ponds.

## Supplementary material

10.1099/acmi.0.000996.v4 Uncited Supplementary Material 1.

## Abbreviations

ANI: average nucleotide identity
ANIb: Average nucleotide identity based on BLASTn
BGCs: biosynthetic gene clusters
CARD: Comprehensive Antibiotic Resistance Database
CRISPR-Cas: Clustered regularly interspaced short palindromic repeats
dDDH: digital DNA–DNA hybridization
GBDP: Genome BLAST Distance Phylogeny
gDNA: genomic DNA
GGDC: Genome-to-Genome Distance Calculator
IMA: integrated mangrove aquaculture
KEGG: Kyoto Encyclopedia of Genes and Genomes
MiGA: Microbial Genomes Atlas
ML: maximum-likelihood
NCBI: National Center for Biotechnology Information
RGI: Resistance Gene Identifier
TYGS: Type (Strain) Genome Server
WSSV: white spot syndrome virus
