# Supplementary material for: Genomic insights into Brevibacterium sediminis strain IMA_C3 isolated from an integrated mangrove aquaculture pond
Source: Access Microbiol. 2026 Feb 13;8(2):000996.v4. doi: 10.1099/acmi.0.000996.v4 (PMC12904602; doi:10.1099/acmi.0.000996.v4)
Supplement: Uncited Supplementary Material 1. [file acmi-8-00996-s001.pdf]

Fig. S1: Circular comparative map of *Brevibacterium sediminis* strain IMA_C3 in comparison with the closest type strains of the genus *Brevibacterium* with GC content and GC skew (+/-)

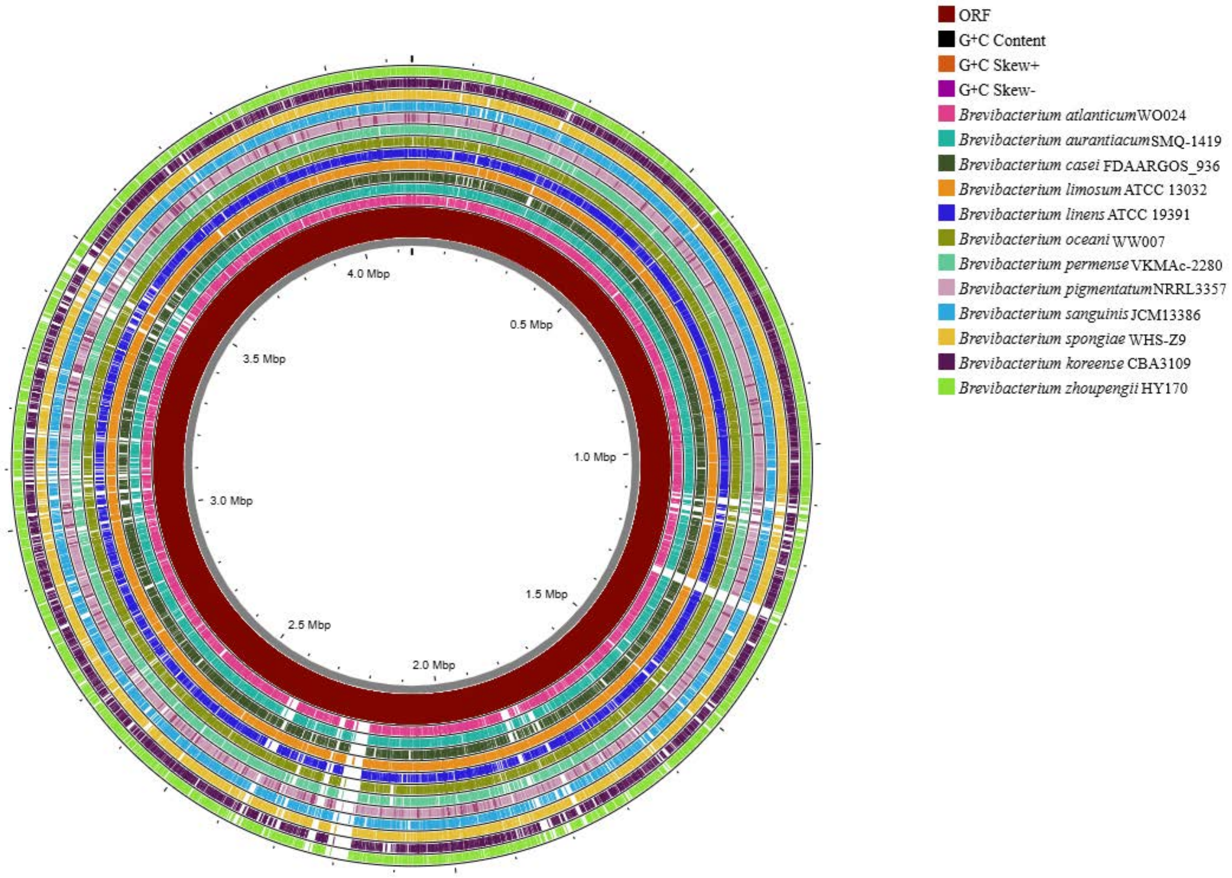

Table S1: List of functional genes present in *Brevibacterium sediminis* strain IMA_C3

KEGG ID	Present genes	Encoded proteins
K00459	*ncd*2, *npd*	nitronate monooxygenase [EC:1.13.12.16]
K01501	*nit*A	nitrilase [EC:3.5.5.1]
K01915	*gln*A, GLUL	glutamine synthetase [EC:6.3.1.2]
K00260	*gud*B, *roc*G	glutamate dehydrogenase [EC:1.4.1.2]
K00261	GLUD1_2, *gdh*A	glutamate dehydrogenase (NAD(P)+) [EC:1.4.1.3]
K00262	*gdh*A	glutamate dehydrogenase (NADP+) [EC:1.4.1.4]
K00265	*glt*B	glutamate synthase (NADPH) large chain [EC:1.4.1.13]
K00847	*scr*K	fructokinase [EC:2.7.1.4]
K00965	*gal*T, GALT	UDPglucose-hexose-1-phosphate uridylyl transferase [EC:2.7.7.12]
K00849	*gal*K	galactokinase [EC:2.7.1.6]
